# DNA methylation regulates TIGIT expression within the melanoma microenvironment, is prognostic for overall survival, and predicts progression-free survival in patients treated with anti-PD-1 immunotherapy

**DOI:** 10.1186/s13148-022-01270-2

**Published:** 2022-04-11

**Authors:** Dennis Niebel, Anne Fröhlich, Romina Zarbl, Simon Fietz, Luka de Vos, Timo J. Vogt, Jörn Dietrich, Judith Sirokay, Pia Kuster, Gonzalo Saavedra, Susana Ramírez Valladolid, Friederike Hoffmann, Sebastian Strieth, Jennifer Landsberg, Dimo Dietrich

**Affiliations:** 1 Department of Dermatology and Allergy, University Medical Center Bonn (UKB), Venusberg-Campus 1, 53127 Bonn, Germany; 2 Department of Otorhinolaryngology, University Medical Center Bonn (UKB), Venusberg-Campus 1, 53127 Bonn, Germany

**Keywords:** T Cell Ig and ITIM Domain (*TIGIT*), DNA methylation, Melanoma, Immunotherapy, Biomarker

## Abstract

**Background:**

TIGIT is an immune checkpoint under investigation as therapeutic target. Understanding the regulation of TIGIT on an epigenetic level might support the development of companion biomarkers.

**Methods:**

We correlated *TIGIT* DNA methylation of single CpG sites with gene expression, signatures of immune infiltrates and interferon-γ, and survival in melanoma. We further analyzed methylation levels in immune cell subsets, melanocyte and melanoma cell lines. *TIGIT* expression patterns within components of the melanoma microenvironment were analyzed by single cell sequencing. We used quantitative methylation-specific PCR, flow cytometry, and immunohistochemistry for correlations between expression and methylation and to assess the effect of pharmacological demethylation of melanoma cells treated with 5‐aza‐2‐deoxycytidine (decitabine). Finally, we investigated the association of patients’ survival with *TIGIT* mRNA and methylation.

**Results:**

Depending on the sequence context of the analyzed CpG site, we found a cell type-specific *TIGIT* gene locus methylation pattern and significant correlations of *TIGIT* methylation with mRNA expression, an interferon γ signature, and distinct immune cell infiltrates, including TIGIT^+^ lymphocytes. We detected a melanoma cell-intrinsic TIGIT protein expression. Pharmacological demethylation of the A375 melanoma cell line led to a constitutive *TIGIT* expression. Low promoter flank methylation and high mRNA expression was associated with patients’ prognosis and predicted progression-free survival in patients treated with anti-PD-1 immunotherapy. A high TIGIT^+^ lymphocyte score was associated with better progression-free survival under anti-PD-1 immunotherapy.

**Conclusions:**

Our data demonstrate an epigenetic regulation of *TIGIT* expression via DNA methylation within the melanoma microenvironment. *TIGIT* DNA methylation and expression may serve as predictive biomarkers in the context of immunotherapies in melanoma.

## Background

Immune checkpoint blockade (ICB) has become an important column in modern oncology. Among different types of malignancies, melanomas generally show a high immunogenicity and overall high response rates to ICB [1]. At this time, the most effective approved ICB therapy for metastatic melanoma is a combination of nivolumab and ipilimumab. However, potentially life threatening or irreversible side effects and both primary and secondary therapy resistance limits its utility. Therefore, it is desirable to identify alternative treatment regimens, which are equally or even more effective but less toxic. One conceivable approach is simultaneous inhibition of the programmed cell death protein 1 (PD-1) axis and of “second generation” immune checkpoints [2]. Multiple agonistic and antagonistic monoclonal antibodies of numerous immune checkpoints are in development or already in clinical use. A possible candidate for combinatorial therapies is the T cell immunoreceptor with Ig and ITIM domains (TIGIT) [3–5]. Only recently, Roche’s monoclonal anti-TIGIT antibody tiragolumab has been granted Breakthrough Therapy Designation (BTD) by the US Food and Drug Administration (FDA) in combination with the anti-PD-L1 antibody atezolizumab for the first-line treatment of metastatic non-small cell lung cancer (NSCLC) with high PD-L1 expression and no genomic *EGFR* or *ALK* aberrations [6]. In addition, several other anti-TIGIT monoclonal antibodies, e.g. BMS-986207 (Bristol Myers Squibb), vibostolimab (Merck Sharp & Dohme), COM902 (Compugen), domvanalimab (Arcus Biosciences), etigilimab (Mereo BioPharma), ociperlimab (BeiGene), IBI939 (Innovent Biologics), and M6223 (Merck KGaA) are in development for treatment of various solid tumors (ClinicalTrials.gov Identifiers: NCT02913313, NCT02964013, NCT04354246, NCT04262856, NCT04047862, NCT04353830, NCT04457778, NCT03628677).

*TIGIT* gene is located on the q-arm of chromosome 3 and encodes a protein of 244 amino acids which acts as a transmembrane receptor in terms of a co-inhibitory immune checkpoint on various immune cells. Its function is best described for T cells and natural killer (NK) cells and is carried out via interaction with nectin 2 (CD112) and poliovirus receptor (CD155, PVR), while affinity is much higher for the latter [7]. DNAX accessory molecule-1 (DNAM-1, CD226) is the co-stimulatory counterpart of TIGIT that interacts with the same ligands; however, TIGIT is capable to superimpose the effect of CD226 in a dose dependent manner in vitro [8]. Effective tumor infiltrating lymphocytes (TILs) activate Th1-associated pathways such as the interferon-γ/Janus kinase/signal transducer and activator of transcription 1 (IFN-γ/JAK/STAT1)–mediated signaling pathway, which leads to increased expression of immune checkpoints on tumor cells [9]. In peritumoral leukocytes, the inhibitory receptor TIGIT is enriched while the costimulatory receptor CD226 is depleted, which results in undermining of an effective cellular anti-tumoral immune response [8]. As a consequence, effector T cell functions, including T cell receptor (TCR) interactions, are impaired. However, the inhibition of apoptosis leads to the maintenance of these “exhausted” cells in the microenvironment [10]. The role of TIGIT in T cell exhaustion is well described in the context of chronic viral infections, e.g. human immunodeficiency virus (HIV) [11, 12]. Other downstream effects include impediment of mitogen-activated protein kinase (MAPK) and phosphoinositide 3-kinase (PI3K) signaling [2] which leads to extenuated NK cell activation in terms of reduced cytotoxicity, granule polarization, and cytokine secretion, including IFN-γ [13, 14]. Another effect of TIGIT is achieved via an inhibitory loop to antigen-presenting cells (APCs) and through promoting functions of regulatory T cells (Tregs) [15]. TIGIT also seems to be involved in polarization of macrophages towards an anti-inflammatory M2 phenotype [16]. On the contrary, TIGIT inhibition is associated with an altered cytokine expression profile, which may pave the way to an IFN-γ driven Th1/Th17 shift predisposing to autoimmune diseases or inflammatory conditions like psoriasis [17].

The role of TIGIT in maintaining immune tolerance by dampening effects of peripheral T cells has been studied widely in mouse models of autoimmunity. Pharmacological TIGIT activation has shown potential to ameliorate murine graft versus host disease (GVHD) [18]. On the other side of the spectrum, TIGIT deficient mice showed retarded progression in different murine tumor models although metastatic spread was not affected [19]. These findings convincingly suggest that high TIGIT expression is involved in the establishment of T cell exhaustion. A most dysfunctional type has been defined by simultaneous overexpression of TIGIT with other co-inhibitory receptors including PD-1 [20]. High levels of these cell subsets within the tumor microenvironment (TME) might be predictive for failure of ICB [21]. Chauvin et al. [22] suggest TIGIT and PD-1 to regulate the expansion and function of tumor antigen-specific T cells and CD8^+^ TILs in melanoma. This is in line with the finding that isolated CD8^+^ T cells of melanoma metastases showed an “exhausted” profile [23]. Moreover, it has been shown that melanoma cells may influence tumor-specific CD8^+^ T cells via TIGIT-CD155 response modulation [24].

NK cell-specific immunosuppressive effects of TIGIT in vitro include the suppression of IFN-γ production via β-arrestin 2-mediated signaling [13]. A pro-inflammatory IFN-γ signature in turn is associated with increased TIGIT expression in immune cells. Consistently, CRISPR-generated *TIGIT* knockout leads to enhanced IFN-γ production, rendering an antitumoral effect in vivo in a murine colorectal cancer model [25]. Taken together, these findings are suggestive of a potentially beneficial therapeutic effect of TIGIT inhibition in metastatic melanoma alone or in combination with PD-1 inhibitors.

The presence of a reversibly exhausted subset of T effector cells in the TME in the course of an inflammatory response may be linked to specific epigenetic alterations [26, 27]. These epigenetic changes include altered histone modification patterns, chromatin structure, DNA methylation of specific gene sites including promoters, and changes in microRNA levels [28]. Methylation of cytosine within the CpG dinucleotide sequence may silence target genes via altered chromatin packing [29]. Moreover, promoter hypermethylation of tumor suppressor genes plays a critical role in many cancer types and is highly relevant in T cell differentiation and T cell exhaustion [30]. It was first reported in 2013 that the activity of Tregs depends on methylation status of *TIGIT* gene in peripheral blood cells of healthy donors [31]. However, until now, knowledge of the epigenetic modifications of immune checkpoint genes in melanoma and corresponding TILs is very limited. Our group repeatedly demonstrated aberrant methylation levels of immune checkpoint genes [i.e. *PD-1*, *PD-L1*, PD-1 ligand 2 (*PD-L2*), *4-1BB*, *LAG3*, and cytotoxic T-lymphocyte associated protein 4 (*CTLA4*)] with a predictive and prognostic value in different malignancies including melanoma [32–38]. The growing number of available therapeutic agents demands reliable predictive biomarkers to identify the most effective treatment modality. Therefore, in the present study we comprehensively analyzed *TIGIT* methylation in melanoma with regard to transcriptional activity, immune cell infiltrates, an IFN-γ signature, markers of T cell activation, patients’ prognosis, and response to immunotherapy.

## Materials and methods

### Patients

We investigated four independent patient cohorts. First, gene methylation data from publicly available data from *N* = 470 primary or metastatic melanomas provided by the TCGA Research Network (http://cancergenome.nih.gov/) was included [39]. We included only primary tumors from patients who provided primary and metastatic tumor tissue. Clinical and cytological (e.g. lymphocyte score) data were obtained from the TCGA Research Network [39]. For a comprehensive analysis of the TME we used RNA-Seq signatures of immune infiltrates as provided by Thorsson et al*.* [40] and Saltz et al*.* [41].

Furthermore, we included two cohorts comprised of *N* = 94 (UKB Non-ICB cohort) and *N* = 43 (UKB ICB cohort) formalin-fixed and paraffin-embedded (FFPE) melanomas from patients treated at the University Medical Center Bonn (UKB). The UKB Non-ICB cohort included primary tumors, cutaneous metastases, and distant metastases from stage I–IV melanoma patients who did not receive immunotherapy. The UKB ICB cohort included primary tumors, distant and cutaneous metastases from stage IV melanoma patients who received anti-PD-1-based ICB (with or without combination with the anti-CTLA-4 antibody ipilimumab) as follows: *N* = 19 received pembrolizumab, *N* = 3 nivolumab, *N* = 1 nivolumab and afterwards pembrolizumab, *N* = 20 ipilimumab / nivolumab immune combination therapy.

Additionally, we included publicly available data from *N* = 128 patient samples from an ICB cohort recently published by Liu et al*.* [42].

Finally, publicly available data from human samples provided by Tirosh et al. [43] and Hannon et al. [44] were included for cell type-specific analyses as described below.

### Cell lines and isolated cells

We included publicly available data from *N* = 59 melanoma cell lines obtained from Gene Expression Omnibus (GEO) accession numbers GSE51547 and GSE68379 (National Center for Biotechnology Information (NCBI), Bethesda, MD, USA). For comparison, we included publicly available data from *N* = 28 leukocyte cell fractions prepared by FACS from healthy donors (GSE103541/GSE166844) [44] and *N* = 3 melanocyte cell lines (GSE44662). Single cell RNA-Seq data from CD45^+^, CD45^−^, T cells, B cells, and macrophages isolated from melanomas were obtained from Tirosh et al. [43].

The human melanoma cell line A375 (RRID:CVCL_0132) was purchased from American Type Culture Collection (ATCC, Manassas, VA, USA). The cell line was authenticated by the Leibniz-Institut DSMZ-Deutsche Sammlung von Mikroorganismen und Zellkulturen GmbH (Braunschweig, Germany) and mycoplasma contamination testing has been performed regularly. A375 cells were grown adherent and maintained in complete RPMI 1640 medium (cat. no. 21875059, Thermo Fisher Scientific, Waltham, MA, USA) supplemented with 10% [v/v] fetal bovine serum (FBS, heat inactivated, cat. no. FBS. S 0615HI, Bio&SELL GmbH, Nuremburg, Germany), 1X MEM (Minimum Essential Medium) Non-Essential Amino Acids Solution (100X stock, cat. no. 11140035, Thermo Fisher Scientific), 1 mM HEPES (1 M stock, cat. no. 15630056, Thermo Fisher Scientific), 1 mM 2-mercaptoethanol (cat. no. 21985023, Thermo Fisher Scientific), 100 U/ml penicillin and streptomycin (10,000 U/ml stock, cat. no. 15140122, Thermo Fisher Scientific), and 1 mM sodium pyruvate (100 mM stock, cat. no. 11360070, Thermo Fisher Scientific). A375 cells were either left untreated for 168 h or treated with demethylating 5‐aza‐2‐deoxycytidine (decitabine, 5‐aza‐dC). For 5‐aza‐dC treatment, 10 μM 5‐aza‐dC (cat. no. ab120842, Abcam, Cambridge, UK) was supplemented to the growth medium every 24 h over a 168 h period.

### Promoter methylation analysis

Gene methylation data (*β* values) generated using the Infinium HumanMethylation450 and EPIC BeadChip (Illumina, Inc., San Diego, CA, USA) were obtained from the TCGA Research Network, GSE51547, GSE68379, GSE103541, and GSE44662, respectively [39, 44]. We included beads that target nine CpG sites within *TIGIT*: cg05943254 (CpG1), cg19440299 (CpG2), cg22577252 (CpG3), cg19421218 (CpG4), cg19456938 (CpG5), cg13669740 (CpG6), cg20832020 (CpG7), cg09246203 (CpG8), and cg22870429 (CpG9). We considered *β* values approximatively as percent methylation.

Methylation analysis of the UKB cohorts was performed using quantitative real-time PCR targeting CpG5. Bisulfite DNA was prepared and quantitative methylation-specific real-time PCR (qMSP) was conducted as described earlier [35] using the following primers and probes (biomers.net GmbH, Ulm, Germany): ggttttttttgtggtttattttatgtagtt (forward primer), aacctacaaaaaacaaatataacctctt (reverse primer), 6-FAM-acctctaaaaaaaaacgatctcgaa-BHQ-1 (probe_methylated_), HEX-ctaaacctctaaaaaaaaacaatctcaaa-BHQ-1 (probe_unmethylated_). The qMSP buffer was prepared as published earlier [45]. For qMSP analysis, we applied the following temperature profile: 20 min at 95 °C and 45 cycles with 15 s at 95 °C, 2 s at 62 °C and 60 s at 58 °C. Relative methylation levels were calculated using cycle threshold (CT) values obtained from probes specifically binding to methylated DNA, respectively, using the following formula: Methylation[%] = 100%/(1 + 2^CTmethylated–CTunmethylated^).

### mRNA expression analysis

mRNA data generated by RNA-Seq were obtained from the TCGA Research Network (TCGA cohort), Liu et al*.* (ICB cohort), and Tirosh et al*.* (single cell sequencing), respectively [39, 42, 43].

RNA-Seq protocols are described in the corresponding publications [39, 42, 43, 46]. In brief, Liu et al*.* used TruSeq RNA Exome (Illumina) technology for the generation of RNA sequencing libraries from degraded FFPE tissue samples that focus on the RNA coding regions and reported Transcripts Per Million (TPM) [42]. Tirosh et al. [43] applied Nextera XT DNA Library Preparation Kit (Illumina) for library preparation from whole transcriptome amplification products as prepared using a modified Smart-Seq2 protocol. Sequencing was performed with an Illumina NextSeq 500 instrument using 30 bp paired-end reads and TPM values were reported [43]. The TCGA Research Network prepared mRNA libraries by means of Illumina TruSeq sample preparation kit and subsequent sequencing using the HiSeq 2000 Sequencing System (Illumina) [39, 46]. The TCGA Research Network provided normalized read counts calculated using the Reads Per Kilobase of exon model per Million mapped reads (RPKM) method [39, 46].

### Immunohistochemistry

In the UKB cohort, we assessed immune cell infiltration and TIGIT protein expression on whole slides by immunohistochemistry (IHC). Briefly summarized, paraffin sections of 4 µm thickness were cut from the tissue block and subsequently stained using the Dako Omnis system (Dako/Agilent Technologies). For IHC, we used mouse monoclonal anti-TIGIT antibody (cat. no. DIA-TG1, Oncodianova GmbH GmbH, Hamburg, Germany; dilution 1:50). We performed heat-induced antigen retrieval with target retrieval solution at pH 6 for 10 min at 117 °C using a steam pressure cooker. The slides were incubated with the primary antibody overnight at 4 °C. Signal detection was performed with an Alkaline Phosphatase Red Detection Kit (Dako/Agilent Technologies, cat. no. K5005). The slides were finally counterstained with hematoxylin and bluing reagent, dehydrated, and mounted. Tonsillar tissue was used as positive control. TIGIT protein expression in the tumor was assessed using the H-score rating the percentage of tumor cells negative (0), weak (1), moderate (2), and strong (3) (H-score: 0–300). Immune cells were scored according to TCGA (lymphocyte score [39]) and TIGIT^+^ immune cells were assessed as percentage fraction from all cells (TIGIT^+^ lymphocyte score).

### Flow cytometry

A375 melanoma cell line pellets were washed with flow cytometry buffer (1X Dulbecco’s Phosphate Buffered Saline [cat. no. 14190094, Thermo Fisher Scientific], 4% [v/v] FBS, 2 mM ethylenediaminetetraacetic acid [EDTA]). 50 µl PerCP/Cyanine5.5 labeled anti-human TIGIT mouse monoclonal antibody (dilution 1:100 in flow cytometry buffer; clone A15153G, RRID:AB_2632933, BioLegend, CA, USA) was added to the cell pellet and incubated for 30 min at 4 °C. The liquid phase was removed and the cell pellet resuspended in 300 µl flow cytometry buffer. Flow cytometry data were acquired with a FACSCanto™ Flow Cytometer (Becton, Dickinson and Company, NJ, USA) and analyzed with FlowJo software (version 10.8.0, Becton, Dickinson and Company).

### Statistical analysis and clinical endpoints

We performed all statistical analyses with SPSS, version 23.0 (SPSS Inc., Chicago, IL). Comparisons of mean values between groups were performed applying one way ANOVA with Bonferroni post-hoc testing and the Wilcoxon-Mann–Whitney *U* test, respectively. We performed Spearman’s *ρ* rank correlation analyses. Significance levels for the Spearman’s *ρ* rank correlation coefficients were computed using a large sample normal theory approximation that utilizes a *t*-distribution. Survival analyses were performed using the Kaplan–Meier method and Cox Proportional Hazards regression using optimized cut-offs for dichotomization of methylation values. Progression-free survival (PFS) was defined as time from ICB start to progression or last follow-up. Overall survival (OS) was defined as time to death or last follow-up. Survival differences between the groups were tested using the log-rank test (Kaplan–Meier) and Wald test (Cox Proportional Hazards). Hazard Ratios (HR) were reported including 95% Confidence Intervals (95%CI). *P* values < 0.05 were considered as statistically significant.

## Results

### *TIGIT* methylation correlates with mRNA expression

**Fig. 1:**
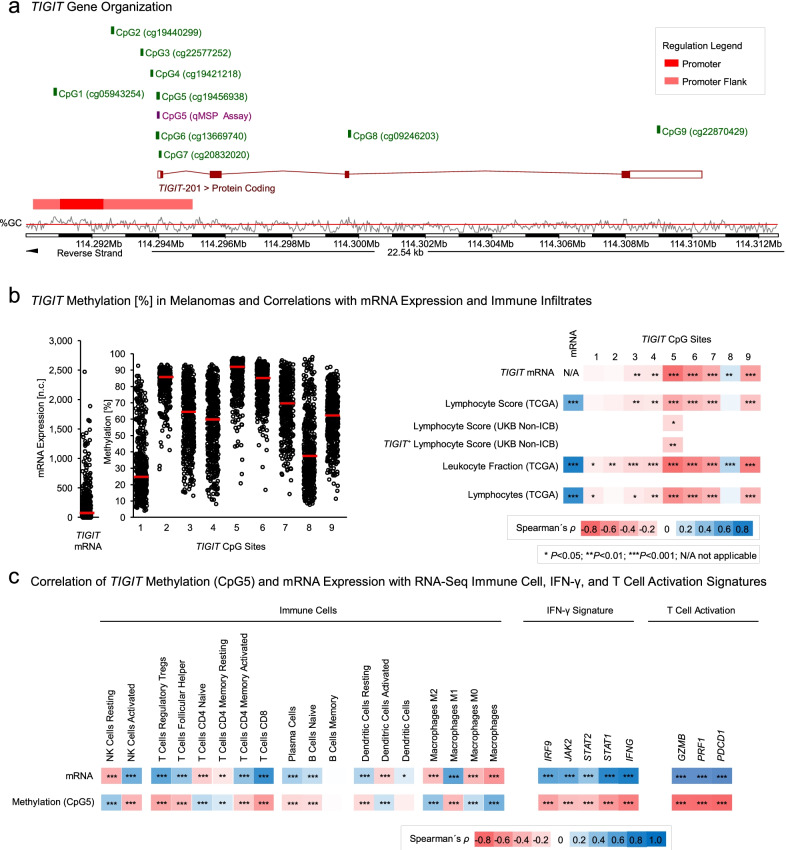
Correlates of *TIGIT* DNA methylation with expression and signatures of immune infiltrates, an IFN-γ signature, and T cell activation markers in melanoma. **a** Genomic organization of the *TIGIT* gene and analyzed loci. Shown are guanosine/cytosine (GC)-density, the *TIGIT* consensus transcript, predicted promoter and its flanks, and target sites of HumanMethylation450 BeadChip beads (CpG1-9) and qMSP assay. The modified illustration was exported from www.ensemble.org (Ensembl Release 103) and is based on Genome Reference Consortium Human Build 38 patch release 13 (GRCh38.p13). **b** Methylation [*β* values] at nine CpG sites (CpG1-9) and correlation with *TIGIT* mRNA expression and with histopathologic lymphocyte score according to TCGA [39] (*N* = 469, TCGA cohort; *N* = 94, UKB Non-ICB cohort), immunohistochemical TIGIT^+^ lymphocyte score (*N* = 94, UKB Non-ICB cohort), methylation signature of leukocyte fraction according to Saltz et al. [41] and lymphocyte RNA-Seq signature according to Thorsson et al. [40]. **c** Spearman’s correlations (Spearman’s *ρ*) of *TIGIT* methylation and mRNA expression with RNA-Seq signatures of immune cell infiltrates (resting and activated NK cells, Tregs, T follicular helper cells, naïve CD4^+^ T cells, resting and activated memory CD4^+^ T cells, CD8^+^ T cells, plasma cells, naïve B cells, memory B cells, resting and activated dendritic cells, and M0/M1/M2 macrophages) and their activation status according to Thorsson et al. [40], IFN-γ (*INFG*, *STAT1*, *STAT2*, *JAK2*, *IRF9*), and cytotoxic markers (*GZMB*, *PRF1*) as well as co-inhibitory markers (*PDCD1*)

**Fig. 2:**
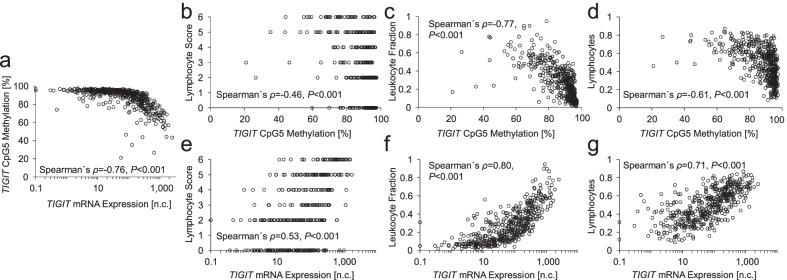
Correlations between *TIGIT* DNA methylation, mRNA expression, and infiltrates. Scatter plots of correlations between *TIGIT* mRNA and CpG5 methylation (**a**), *TIGIT* CpG5 methylation and lymphocyte score (according to TCGA [39]; **b**), *TIGIT* CpG5 methylation and leukocyte fraction (according to Saltz et al. [41]; **c**), *TIGIT* CpG5 methylation and lymphocytes (according to Thorsson et al. [40]; **d**), *TIGIT* mRNA expression and lymphocyte score (**e**), *TIGIT* mRNA expression and leukocyte fraction (**f**), and *TIGIT* mRNA expression and lymphocytes (**g**). Expression levels of 0 n.c. were set to 0.1 n.c. in order to allow for a logarithmic illustration. Shown are Spearman’s *ρ* rank correlation coefficients and *P* values

We analyzed nine CpG sites within the *TIGIT* promoter flanks (CpG1-7), within the gene body (CpG8), and within the 3’UTR (CpG9; Fig. 1a) in melanomas from the TCGA cohort. Overall, mean methylation levels (*β*-values) of the analyzed loci ranged from 31% (CpG1) to 87% (CpG5; Fig. 1b). Next, we analyzed the correlation of *TIGIT* methylation levels with mRNA expression to investigate a possible epigenetic regulation. Methylation levels of CpG3–CpG7 and CpG9, located within the promoter flanks and 3’UTR, showed a strong and statistically significant inverse correlation with *TIGIT* mRNA expression, whereas methylation of CpG8 located within the gene body showed a significant positive correlation (Fig. 1b). CpG5 methylation showed the strongest inverse correlation with *TIGIT* mRNA expression (Spearman’s *ρ* = − 0.76, *P* < 0.001; Figs. 1b, 2a).

### *TIGIT* methylation correlates with signatures of immune cell infiltrates

**Fig. 3:**
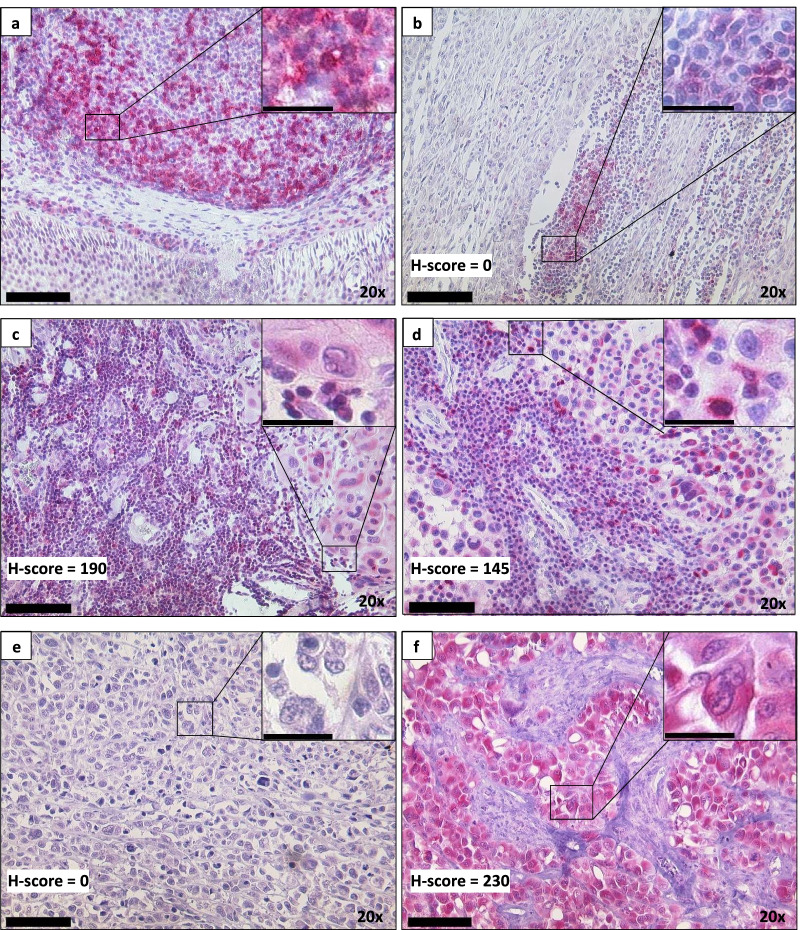
TIGIT protein expression in tonsillar and melanoma tissue. Representative immunohistochemical staining patterns in a tonsil (**a**) and various melanomas (**b–f**). **a** In tonsillar tissue (positive control), lymphatic germinal centers stain strongly positive in proximity to the mantle zone. **b** Melanoma with TIGIT^+^ TILs and TIGIT^−^ tumor cells (H-score = 0). **c, d** Melanomas with dense immune cell infiltrate; both tumor and immune cells weakly/moderately positive for TIGIT expression (**c**: H-score = 190, **d**: H-score = 145). **e** Melanoma with TIGIT^−^ TILs and TIGIT^−^ tumor cells (H-score = 0). **f** Melanoma with strong TIGIT expression in the majority of tumor cells (H-score = 230). **a-f** Original magnification 200×, scale bar indicates 100 µm in the main photograph; scale bar indicates 25 µm in the magnified areas

Differential *TIGIT* DNA methylation levels among individual tumors might be caused by the composition of the TME, i.e. the proportion of infiltrating immune cells. We tested this hypothesis by correlation of *TIGIT* mRNA expression and methylation levels with signatures of immune cell infiltrates. In the TCGA cohort, we found significant inverse correlations between methylation of the promoter flank (CpG3-7) and the 3’UTR (CpG9) with histopathologic lymphocyte score (Fig. 1b). Again, CpG5 methylation showed the strongest inverse correlation (Spearman’s *ρ* = − 0.46, *P* < 0.001; Figs. 1b, 2b). Accordingly, mRNA levels correlated positively with lymphocyte score (Spearman’s *ρ* = 0.53, *P* < 0.001; Figs. 1b, 2e). This pattern of correlations was concordant to correlations with leukocyte fraction and lymphocyte infiltrates as previously computed by Thorsson et al. [40] and Saltz et al. [41] based on RNA-Seq and methylation signatures, respectively (Figs. 1b, 2c, d, f, g). In order to confirm our findings, we developed a qMSP assay targeting CpG5 which showed a strong negative correlation of DNA methylation with mRNA expression and immune cell infiltration. We tested a cohort of patients of our department (*N* = 94, UKB Non-ICB cohort) and confirmed a weak but statistically significant negative correlation between *TIGIT* gene methylation and lymphocyte score (Spearman’s *ρ* = − 0.24, *P* = 0.021; Fig. 1b). Next, we investigated the correlation between methylation levels and TIGIT expression of immune cells on the protein level. We established a TIGIT immunohistochemistry using lymphatic tissue (tonsil). A subset of immune cells in lymphatic germinal centers in proximity to the mantle zone stain strongly positive (Fig. 3a). TIGIT^+^ immune cells were found in most cases of melanomas, in which tumor infiltrating lymphocytes were present (Fig. 3b–d). From FFPE tissue sections, we determined a TIGIT^+^ lymphocyte score as defined as the portion of TIGIT^+^ immune cells from the total number of cells. *TIGIT* CpG5 methylation showed a weak but significant negative correlation with TIGIT^+^ lymphocyte score (Spearman’s *ρ* = − 0.27, *P* = 0.009). We confirmed the significant negative correlations between *TIGIT* gene methylation and lymphocyte score (Spearman’s *ρ* = − 0.46, *P* = 0.003) and with TIGIT^+^ lymphocyte score (Spearman’s *ρ* = -0.59, *P* < 0.001) in a small cohort of *N* = 43 ICB-treated melanoma patients (UKB ICB cohort, *N* = 38 samples with available TIGIT IHC). Of note, we detected TIGIT protein expression in a number of melanoma cells (Figs. 3b–d, 2f). We investigated this finding in more detail as described below.

### *TIGIT* methylation shows a differential pattern among cell types and corresponds to mRNA expression levels

**Fig. 4:**
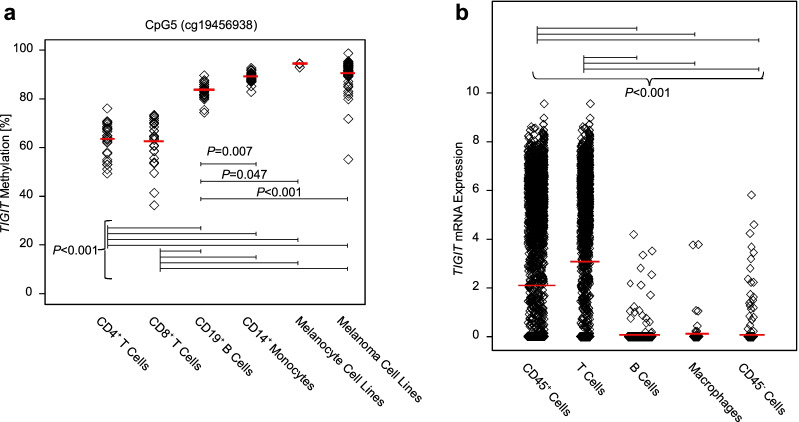
Cell type-specific *TIGIT* methylation (CpG5) and mRNA expression. **a** *TIGIT* methylation at CpG5 in isolated leukocytes (CD4^+^ T cells [mean methylation: 63.5% [95% CI 60.9–66.2]], CD8^+^ T cells [62.6% [95% CI 58.9–66.3]], B cells [83.8% [95% CI 82.5–85.1]], and monocytes [89.47% [95% CI 88.6–90.3]]) from *N* = 28 healthy donors, melanocyte (*N* = 3, 94.8% [95% CI 92.8–96.8]) and melanoma cell lines (*N* = 59, 90.8% [95% CI 89.2–92.5]). **b** Single cell *TIGIT* mRNA expression of different cell subtypes (CD45^−^ [mean expression: 0.0391 TPM [95% CI 0.0202–0.0581], *N* = 1257 and CD45^+^ cells [2.0649 TPM [95% CI 1.9684–2.1605]], *N* = 3,256; T cells [3.0444 TPM [95% CI 2.9167–3.1720]], *N* = 2068; B cells [0.0595 TPM [95% CI 0.0269–0.0920]], *N* = 515; macrophages [0.0860 TPM [95% CI − 0.0005 to 0.1726]], *N* = 126) from 19 freshly procured human melanoma samples as previously reported by Tirosh et al. [43]. Bars indicate significant differences (*P* < 0.05, ANOVA with Bonferroni post-hoc test)

Our results strongly suggested a differential methylation pattern between components of the TME. In order to test this hypothesis, we analyzed CpG5 methylation levels in melanoma cell lines (*N* = 59), cell lines of melanocytes (*N* = 3), and peripheral leukocytes (monocytes, B cells, CD8^+^ and CD4^+^ T cells) from healthy donors (*N* = 28). Statistical analysis revealed highly significant differences of CpG5 methylation among the different cell subsets (*P* < 0.001, ANOVA; Fig. 4a). Overall, immune cells showed significantly lower methylation levels when compared to melanocytes and melanoma cells. Accordingly, we detected significantly higher *TIGIT* mRNA expression levels in CD45^+^ leukocytes compared to CD45^−^ non-immune cells (Fig. 4b). However, elevated mRNA expression levels were also present in a subset of CD45^−^ non-immune cells. Among the immune cell subsets, higher methylation levels were present in B cells and monocytes compared to CD4^+^ and CD8^+^ T cells (Fig. 4a). CD4^+^ and CD8^+^ T cells, however, did not exhibit significantly different methylation levels (*P* = 1.0, ANOVA with Bonferroni post-hoc test). Again, these findings were consistent with mRNA expression levels: *TIGIT* expression was significantly higher in T cells compared to macrophages and B cells (Fig. 4b).

### *TIGIT* methylation correlates with immune cell activation status, an IFN-γ signature, and T cell activation markers

*TIGIT* knock-down in T cells results in upregulation of IFN-γ [47]. Therefore, we analyzed *TIGIT* mRNA expression and methylation at CpG5 within distinct immune cell lineages with regard to activation status and IFN-γ/JAK/STAT1 pathway–associated genes (Fig. 1c). *TIGIT* mRNA was positively associated with signatures of numerous pro-inflammatory immune cell subsets, including M1 macrophages, activated dendritic cells, CD8^+^ T cells and activated NK cells. In line with this finding, *TIGIT* mRNA showed a significant positive correlation with the mRNA of interferon γ and the IFN-γ–regulated genes signal transducer and activator of transcription 1 and 2 (*STAT1* and *STAT2*), Janus kinase 2 (*JAK2*), and interferon regulatory factor 9 (*IRF9*; *IFN-γ*: Spearman’s *ρ* = 0.89, *STAT1*: *ρ* = 0.75, *STAT2*: *ρ* = 0.37, *JAK2*: *ρ* = 0.55, and *IRF9*: *ρ* = 0.57; all *P* < 0.001, *N* = 468). Moreover, genes associated with cytotoxic activity of T cells and NK cells, including perforin 1 (*PRF1*; Spearman’s *ρ* = 0.91, *P* < 0.001) and granzyme B (*GZMB*; Spearman’s *ρ* = 0.87, *P* < 0.001), were found to be positively correlated with *TIGIT* mRNA. Strikingly, high mRNA expression of programmed cell death protein 1 (*PD-1*, *PDCD1*) also correlated with high expression of *TIGIT* (Spearman’s *ρ* = 0.94, *P* < 0.001). For all aforementioned immune cell subsets and genes, an inverse correlation was found for *TIGIT* CpG5 methylation. The corresponding anti-inflammatory counterparts, namely M2 macrophages and resting dendritic cells, showed opposing correlations both of mRNA and CpG5 methylation. Divergently, a robust positive correlation for *TIGIT* mRNA could be demonstrated for Tregs.

### Pharmacological demethylation induces TIGIT expression in melanoma cells

**Fig. 5:**
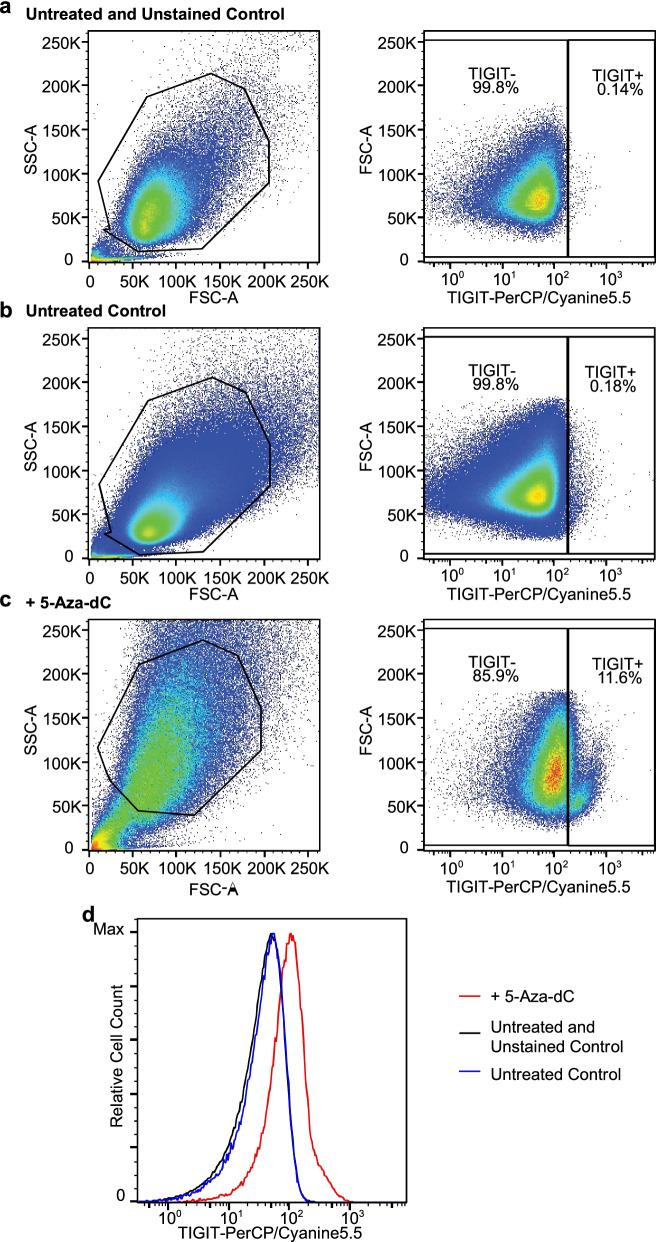
Flow cytometry analysis of TIGIT expression of pharmacologically demethylated melanoma cells. Representative SSC/FSC and FSC/TIGIT-PerCP/Cyanine5.5 dot plots of untreated and unstained (**a**), untreated (**b**), and 5‐aza‐dC treated A375 melanoma cells (**c**). **d** Flow cytometric histograms showing TIGIT expression of pharmacologically demethylated (5‐aza‐dC treated) compared to untreated A375 melanoma cell line

Since we found *TIGIT* mRNA expression in CD45^−^ cells from melanoma tissues (Fig. 4b) and TIGIT protein expression in melanoma cells via IHC (Fig. 3c–d, f), we sought to analyze tumor cell-intrinsic TIGIT expression in more detail. Tumor cells showed either none or moderate to strong TIGIT expression in a rather cytoplasmatic pattern. Although some melanoma samples displayed negative and positive tumor cells in different areas within one section, most samples showed consistent negativity. We used the H-scoring system to quantify TIGIT protein expression in the UKB Non-ICB cohort and correlated it with methylation levels determined by qMSP targeting CpG5. However, there was no significant negative correlation to H-score (Spearman’s *ρ* = 0.14; *P* = 0.18). We further investigated *TIGIT* mRNA expression and the effect of the hypomethylating agent 5‐aza‐dC in the A375 melanoma cell line. As shown in Fig. 5, flow cytometry analysis revealed absence of TIGIT expression in untreated A375 cells and elevated expression after pharmacological demethylation.

### *TIGIT* mRNA expression and methylation predicts survival in ICB and non-ICB treated melanoma patients

**Fig. 6:**
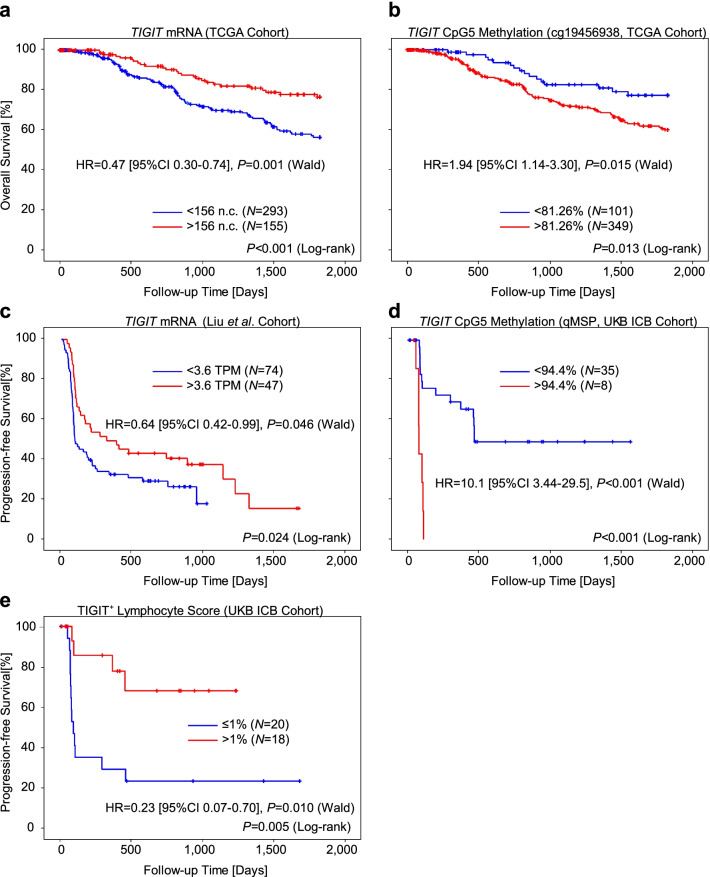
Kaplan–Meier analysis of overall and progression-free survival in melanoma patients stratified according to *TIGIT* mRNA expression, CpG5 methylation, and TIGIT^+^ lymphocyte score. **a** Overall survival in melanoma patients stratified according to tumor *TIGIT* mRNA expression levels (TCGA cohort). **b** Overall survival in melanoma patients stratified according to tumor *TIGIT* methylation levels at CpG5 (TCGA cohort). **c** Progression-free survival in ICB treated melanoma patients stratified according to tumor *TIGIT* mRNA expression levels (Liu et al. cohort [42]). **d, e** Progression-free survival in ICB treated melanoma patients stratified according to tumor *TIGIT* methylation levels at CpG5 (**d**) and TIGIT^+^ lymphocyte score (**e**) (UKB ICB cohort). All *P* values refer to log-rank tests

Since we found significant correlations between *TIGIT* mRNA and methylation levels with known prognostic and predictive factors, i.e. immune cell infiltrates and IFN-γ signature, we performed survival analyses of melanoma patients stratified according to *TIGIT* mRNA and methylation levels. First, we tested the prognostic value in melanoma patients included in the non-ICB TCGA cohort. Patients with high *TIGIT* mRNA expressing tumors above the cutoff (156 n.c.) had a longer overall survival compared to patients with low expressing tumors (HR = 0.47 [95%CI 0.30–0.74], *P* = 0.001; Fig. 6a). Concordantly, patients with *TIGIT* methylation above the cutoff (81.26%) had poorer outcome compared to patients with hypomethylated tumors (HR = 1.94 [95%CI 1.14–3.30], *P* = 0.015; Fig. 6b). Although overall survival data was only available from *N* = 31 melanoma patients included in the UKB Non-ICB cohort, we were able to confirm the poor outcome of patients with hypermethylated tumors (HR = 4.96 [95%CI 1.03–24.0], *P* = 0.047, cutoff: 82%, *N* = 16 < cutoff, *N* = 15 > cutoff). We did not detect significant associations between overall survival and TIGIT^+^ lymphocyte score or TIGIT expressing tumor cells (H-score). However, the small number of patients with available overall survival data limits the validity of this result. We further analyzed *TIGIT* mRNA with regard to progression-free survival in an ICB cohort comprised of *N* = 128 patients recently published by Liu et al. [42]. *TIGIT* mRNA expression above the cutoff (3.6 TPM) was significantly associated with longer progression-free survival (HR = 0.64 [95%CI 0.42–0.99], *P* = 0.046; Fig. 6c). Accordingly, we found prolonged progression-free survival in patients with hypomethylated melanomas in a small cohort of *N* = 43 ICB-treated patients (UKB ICB cohort; HR = 10.1 [95%CI 3.44–29.5], *P* < 0.001, cutoff: 94.4%; Fig. 6d). Finally, we analyzed TIGIT^+^ lymphocyte score and TIGIT H-score with regard to progression-free survival in the UKB ICB cohort. TIGIT IHC staining was available from *N* = 38 tumors. Patients with TIGIT^+^ lymphocyte scores > 1% had a significant better progression-free survival compared to patients with TIGIT^+^ lymphocyte scores ≤ 1% (HR = 0.23 [95%CI 0.07–0.70], *P* = 0.010; Fig. 6e). We did not find a significant association between H-score and progression-free survival. Again, this negative result might be attributable to the small sample size.

## Discussion

Little is known about the epigenetic regulation of TIGIT which evolves as important stakeholder among the variety of “second generation” immune checkpoints. In this study, we investigated a potential epigenetic regulation of TIGIT expression via DNA methylation and tested whether *TIGIT* gene methylation might serve as a feasible biomarker in melanoma. Due to the implementation of high performance DNA methylation analyses platforms, i.e. Infinium technology, it is nowadays a generally accepted concept that DNA methylation highly depends on the sequence context of specific CpG sites. Hence, gene-specific methylation analyses require a thorough view at single CpGs with regard to their position within the gene, e.g. promoter, promoter flank, and gene body. In the present study, we exploited the Infinium technology to compile a detailed picture of the *TIGIT* methylation landscape in melanoma.

We investigated nine single CpG sites within the *TIGIT* promoter and gene body in melanomas from the TCGA cohort, isolated immune cells from peripheral blood, melanoma and melanocyte cell lines. We identified methylation of a specific CpG site, referred to as CpG5, of high significance within the melanoma TME. This CpG is located in the promoter flank in ultimate proximity to the transcription start site of the main *TIGIT* transcript. CpG5 methylation correlated inversely with *TIGIT* mRNA expression and TIGIT^+^ lymphocyte infiltrates in melanoma. We found significantly different methylation levels among subsets of isolated peripheral blood leukocytes, melanoma cell lines and between isolated lymphocytes which underscores the previously reported results of demethylation-dependent activity in peripheral lymphocytes [31]. Lowest methylation levels were present in T cells compared to melanoma cells, melanocytes, B cells, and monocytes. B cells revealed a lower methylation level compared to monocytes, melanoma cells, and melanocytes. However, we did not find significant differences between monocytes, melanoma cells, and melanocytes. The inverse correlation between methylation and mRNA expression and TIGIT^+^ lymphocytes on the one hand and the methylation differences between cell types on the other hand suggest CD45^+^ cells, including T cells, to be the main source of *TIGIT* mRNA expression in melanoma compared to B cells, monocytes/macrophages, and CD45^−^ tumor cells. We confirmed this hypothesis using single cell RNA-Seq data from melanomas provided by Tirosh et al. [43]. Accordingly, significant inverse correlations between CpG5 methylation and immune cell infiltrates were present in melanomas from the TCGA cohort; a finding that we validated in an independent melanoma cohort. Furthermore, we detected significant positive as well as negative correlations between CpG5 methylation and signatures of distinct immune cell infiltrates, including TIGIT^+^ lymphocytes. Of note, the direction of the correlations depended rather on the activation status of the specific immune cell lineage than on the type of immune cell lineage. For example, high amounts of *TIGIT* mRNA were associated with both pro-inflammatory subsets of immune cells and anti-inflammatory Tregs. These results reflect the complex simultaneous dynamic interplay between pro-inflammatory and anti-inflammatory signals within the TME and point towards a context-dependent expression of immune-checkpoints in different immune cells.

We further found significant inverse correlations between CpG5 methylation and an IFN-γ signature (*IFNG*, *STAT1*, *STAT2*, *JAK2*, and *IRF9* mRNA expression [48]) and T cell activation markers (granzyme B, perforin 1, and PD-1 mRNA expression [49]). Generally, an IFN-γ signature within the TME is regarded as a favorable predictive biomarker in a variety of cancers [50]. On the protein level we expected high expression of TIGIT in immune cells in the analyzed melanoma samples [10] and confirmed the presence of a subset of TIGIT^+^ TILs. Concordantly, we identified a significant inverse correlation between TIGIT^+^ TILs and CpG5 methylation. The corresponding highly significant positive correlation of *PD-1* and *TIGIT* mRNA expression is in line with an “exhausted” T cell subset as previously identified [22].

Remarkably, we also found a significant fraction of melanoma cells that express TIGIT protein via IHC. We considered unspecific staining via diffusion as one explanation, however, the staining was found to be specific in a cytoplasmatic pattern, which is compatible with a transmembrane protein. However, TIGIT protein expression did not correlate with CpG5 methylation, suggesting a more complex TIGIT regulation in melanoma cells compared to immune cells. To further reappraise our assumption of an epigenetic regulation of TIGIT expression, we performed cell culture experiments with a human melanoma cell line. TIGIT protein expression was increased after an unspecific global demethylation using 5‐aza‐dC. Our results potentially imply that malignant cells with primary or acquired resistance to ICB might be responsive to ICB in combination with demethylating agents like 5‐aza‐dC which are widely available.

Finally, we investigated the clinical relevance of *TIGIT* methylation, mRNA expression, and TIGIT^+^ lymphocyte infiltration with regard to survival in melanoma patients. We found a significant association of *TIGIT* CpG5 hypomethylation and mRNA overexpression with prolonged overall survival in the TCGA cohort and progression-free survival in anti-PD-1 treated melanoma patients. Moreover, high TIGIT^+^ lymphocyte infiltration was associated with better progression-free survival. However, the small sample sizes of the analyzed cohorts is a limitation of the study and requires further validation in independent sample sets.

The significance of epigenetics in the context of mechanisms of resistance to ICB increasingly receives attention. Epigenetic changes affect immune infiltrates as well as tumor cells, however, with different consequences. On the one hand, an IFN-γ-induced and epigenetic-driven dedifferentiation of melanoma cells has been reported to be associated with response to ICB [51]. On the other hand, resistance to ICB might be associated with a stable terminally exhausted T cell phenotype that is epigenetically determined and prevents ICB-mediated reinvigoration of T cell cytotoxicity [26, 27]. Among epigenetic mechanisms, de novo DNA methylation significantly associates with T cell exhaustion, resulting in resistance to ICB [52]. These findings support the promise of epigenetics, in particular DNA methylation, as a source for the development of predictive biomarkers for ICB. So far, mostly universal biomarkers are established to predict response to ICB (e.g. tumor mutational burden and immune cell infiltrates). However, the increasing number of targets in ICB, including “second generation” co-inhibitory receptors like TIGIT, requires more specific biomarkers. Our group previously demonstrated that other immune checkpoints, i.e. CTLA-4 [36], 4-1BB [34], and LAG3 [35], are also epigenetically regulated via DNA methylation. We therefore propose to screen for hypomethylation or hypermethylation of certain target genes in material obtained from metastases to identify an “exhausted” TIL profile. This is further justified as a group of researchers proposed a most dysfunctional phenotype of TILs, which show a co-expression of TIGIT, LAG3, and PD-1 in CD8^+^ T cells [19]. These patients almost certainly require a combinatorial therapy to overcome T cell exhaustion, which might consist of a combination of PD-1 blockade with TIGIT or LAG3 blockade. As the CD226–TIGIT axis is centrally involved in the Th1/Th17 balance in cancer and autoimmunity, possible side effects of TIGIT blockade include exacerbation of autoimmune diseases similar to combined PD-1/CTLA-4 blockade [53]. As the oncologic pipeline continues to grow diverse in many indications, it will be crucial to enable the best possible allocation of patients to suitable clinical trials. Taken together, our present data shows the strong association of *TIGIT* CpG5 methylation with immunologic features that are known to predict response to ICB. While we were able to indicate a prognostic value of *TIGIT* methylation and mRNA expression even in ICB-treated melanomas, it is not clear yet, if *TIGIT* methylation and mRNA expression has the potential to predict response to ICB or if it is rather only prognostic under ICB treatment. However, our results warrant the further analysis of *TIGIT* methylation and mRNA expression in ICB-treated patients, particularly in patients treated with TIGIT-directed ICB once such cohorts are available. Giving an outlook it will be helpful to tailor immunotherapies to the patient individually based on analyses of genome, epigenome, transcriptome, and microbiome [54].

In conclusion, our results suggest that *TIGIT* mRNA expression is regulated via gene methylation. In addition, we observed a positive correlation between *TIGIT* DNA methylation with known overall survival and molecular features of immune response, including TIGIT^+^ lymphocyte infiltration, which provides early evidence of *TIGIT* methylation as a potential prognostic or even predictive biomarker in melanoma patients.

## Data Availability

The datasets used in the current study are available from the corresponding author on reasonable request, from TCGA Research Network (http://cancergenome.nih.gov/) [39], from studies by Tirosh et al. [43], Thorsson et al. [40], Saltz et al. [41], and Liu et al. [42] or can be downloaded from Gene Expression Omnibus (GEO Accession: GSE51547, GSE68379, GSE103541, GSE166844, GSE44662).
